# Age and the Residual Risk of Cardiovascular Disease following Low Density Lipoprotein-Cholesterol Exposure

**DOI:** 10.3390/biomedicines11123208

**Published:** 2023-12-02

**Authors:** Carola S. König, Amar Mann, Rob McFarlane, John Marriott, Malcolm Price, Sudarshan Ramachandran

**Affiliations:** 1 Department of Mechanical and Aerospace Engineering, Brunel University London, London UB8 3PH, UK; 2 Institute of Clinical Sciences, University of Birmingham, Birmingham B15 2TT, UK; amar.mann@uhb.nhs.uk (A.M.); rjm910@student.bham.ac.uk (R.M.); j.f.marriott@bham.ac.uk (J.M.); 3 Institute of Applied Health Research, University of Birmingham, Birmingham B15 2TT, UK; m.price.2@bham.ac.uk; 4 Department of Clinical Biochemistry, University Hospitals Birmingham NHS Foundation Trust, Birmingham B15 2GW, UK; 5 Department of Clinical Biochemistry, University Hospitals of North Midlands, Staffordshire ST4 6QG, UK; 6 School of Medicine, Keele University, Staffordshire ST5 5BG, UK

**Keywords:** mathematical modelling, residual cardiovascular risk, age and cardiovascular disease, low density lipoprotein cholesterol exposure, non-LDL-C cardiovascular risk factors

## Abstract

We believe that there is sufficient evidence from basic science, longitudinal cohort studies and randomised controlled trials which validates the low-density lipoprotein cholesterol (LDL-C) or lipid hypothesis. It is important that we can communicate details of the cardiovascular disease (CVD) risk reduction that the average patient could expect depending on the scale of LDL-C decrease following lipid lowering therapy. It is also essential that residual risk (ResR) of CVD be highlighted. To achieve this aim by using existing trial evidence, we developed mathematical models initially for relative risk reduction (RRR) and absolute risk (AR) reduction and then showed that despite optimising LDL-C levels, a considerable degree of ResR remains that is dependent on AR. Age is significantly associated with AR (odds ratio: 1.02, 95% confidence intervals: 1.01–1.04) as was previously demonstrated by analysing the Whickham study cohort using a logistic regression model (age remaining significant even when all the other significant risk factors such as sex, smoking, systolic blood pressure, diabetes and family history were included in the regression model). A discussion of a paper by Ference et al. provided detailed evidence of the relationship between age and AR, based on lifetime LDL-C exposure. Finally, we discussed non-traditional CVD risk factors that may contribute to ResR based on randomised controlled trials investigating drugs improving inflammation, thrombosis, metabolic and endothelial status.

## 1. Introduction

Cardiovascular disease (CVD) owing to atherosclerosis is globally one of the most common causes of mortality with the World Health Organisation (WHO) estimating that 17.9 million lives are lost each year to CVD [1]. Although CVD rates have been decreasing in Western Europe, they still remain high; the rates in 2019 with median (interquartile range, IQR) values per 100,000 individuals were 469.0 (459.9–480.4), 5249 (5006–5391), and 196.2 (183.3–228.8) for incidence, prevalence and age standardized mortality rate, respectively [2].

Various risk factors for CVD, both modifiable and non-modifiable, have been identified from prospective studies such as the Framingham Heart Study, the prospective cardiovascular Münster (PROCAM) study, the Systematic COronary Risk Evaluation (SCORE project) with SCORE2 and SCORE2-OP algorithms published recently and QRISK based on the UK QRESEARCH database; these risk factors include age, male gender, elevated blood pressure, diabetes, smoking, dyslipidaemia and others [3,4,5,6,7,8]. In the United Kingdom, the National Institute for Health and Care Excellence (NICE) guidelines suggest that primary care uses the QRISK risk calculator, an algorithm including a combination of traditional risk factors (age, blood pressure, cholesterol etc.) and newer variables (family history, body mass index, erectile dysfunction and ethnicity etc.) with treatment thresholds based on a predicted absolute CVD risk [8].

The data for this present review originated from a 2020 narrative review through a PubMed search for CVD risk algorithms and randomised controlled trials using LDL-C reducing agents (all the statin trials had been included in the CTT collaboration) [9]. A search (using PubMed) was conducted to identify any publications with CVD risk reduction and randomised controlled trials (RCTs) as keywords. Papers that described randomised controlled trials examining CVD risk reduction via non-LDL-C reducing agents were selected. Any trial attributing the CVD risk reduction to LDL-C reduction was excluded. Papers addressing RCTs that were considered to be of interest regarding mechanisms that may contribute to residual risk (ResR) were selected and described in this review.

Absolute risk (AR) is the risk at a one very specific time point during follow-up (e.g., after 5 years of follow-up). Age is the most important risk factor of AR in every CVD predictive algorithm [3,4,5,6,7,8].

The above-mentioned algorithms are not recommended in individuals at high risk of CVD such as those with established CVD, peripheral vascular disease, familial hypercholesterolaemia (FH) and aortic aneurysm (secondary prevention) [9]. Heterozygous FH is an inherited monogenic disease (prevalence ranging from 0.25–0.52%) [10], and without low density lipoprotein cholesterol (LDL-C) lowering therapy, around 50% of men and 30% of women are seen to develop coronary heart disease by the age of 60 years [11]. Hence, age does not play a part in treatment decisions in these high-risk patients. There is some debate whether CVD is best reduced with LDL-C reducing agents by using AR thresholds such as the QRISK algorithms or life-time risk reduction, which suggests earlier lipid lowering to prevent individuals attaining a high risk [12].

The aim of this brief review is to develop methods that can be used to communicate CVD risk indices to patients. This would take into account AR, absolute risk reduction (ARR), relative risk reduction (RRR) and finally ResR, as well as how these indices vary with the age of the patient. Following this, we briefly review possible mechanisms which may contribute to ResR.

## 2. Review of the Lipid Hypothesis

The LDL-C (or lipid) hypothesis is based on randomised controlled trials (RCTs) demonstrating associations between LDL-C reduction with agents such as resins, statins, ezetimibe and more recently Proprotein Convertase Subtilisin/Kexin Type-9 inhibitors (PCSK9) and a decrease in CVD [9]. This review details all the RCTs that provided evidence validating the lipid hypothesis and will not be repeated in this review. The Lipid Research Clinics Coronary Primary Prevention Trial demonstrated a reduction of CVD following LDL-C reduction with the resin, cholestyramine, pointing initially at the lipid hypothesis [13]. Pivotal evidence for the lipid hypothesis came from the Scandinavian Simvastatin Survival Study in 1994, which was subsequently confirmed by numerous RCTs as seen in the Cholesterol Treatment Trialists’ (CTT) Collaboration [14]. It appeared that each subsequent study showed benefit in lower-risk cohorts with varying characteristics. The CTT collaboration included 5 trials (39,612 patients) comparing greater vs. lesser efficacious statins (either in type or dose) and 21 trials (129,526 patients) comparing statins with placebo [14]. Both types of RCTs were combined and a similar relative risk reduction (RRR) of 22% (rate ratio = 0.78) in CVD per 1.0 mmol/L LDL-C reduction was observed (rate ratio: 0.78, 95% CI: 0.76–0.80; *p* < 0.0001) [14]. RRR is the ratio of two absolute risks. Hence, a rate ratio of 0.78 indicates that the incidence rate is reduced by 22% or also that the risk is reduced by 22%. Further, non-statin trials with ezetimibe (SHARP, IMPROVE-IT) [15,16] and Proprotein Convertase Subtilisin/Kexin Type-9 inhibitors (FOURIER, ODESSEY) [17,18], lowering LDL-C via different mechanisms, also reduced CVD risk, with the decrease being in keeping with the RRR observed in the CTT Collaboration [14]. It is important to note that although statins have been seen to reduce CVD in both primary and secondary prevention, rate ratios in statin trials are often lower in primary prevention trials than in secondary prevention trials. However, this was not evident in the CTT Collaboration; rate ratios (95% confidence intervals) were 0.79 (0.76–0.82), 0.81 (0.71–0.92) and 0.75 (0.69–0.82) in individuals with coronary heart disease, vascular disease other than coronary heart disease and no CVD, respectively [14].

Despite optimizing CVD risk management, a significant risk of CVD remains, i.e., ResR. Thus, it is essential to gain an understanding of factors associated with ResR to further reduce CVD events. In this paper we examine the association between age and ResR.

## 3. Calculation of Residual Risk

We use the accrued evidence that led to the lipid hypothesis to calculate ResR. The CTT collaboration, which is widely quoted, was a comprehensive review of the statin RCTs with rate ratios of the subgroups (apart from age categories which is addressed in a subsequent section) very similar to the overall rate ratio [14].

**Figure 1:**
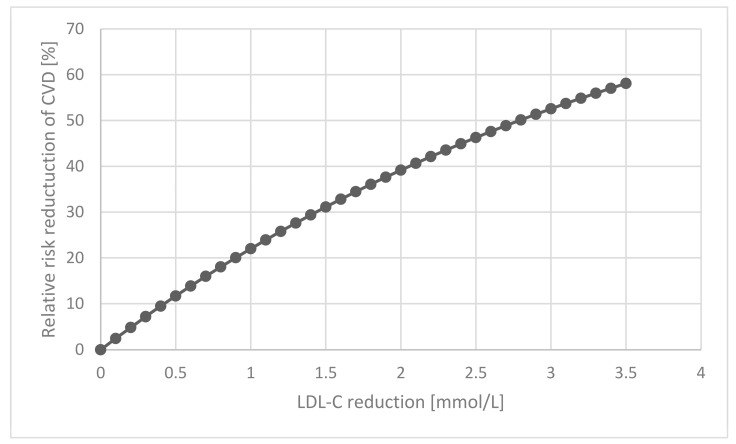
The association between Relative Risk Reduction and LDL-C reduction. This is based on Equation (1), using the results of the CTT collaboration, where the overall rate ratio was 0.78 (95% confidence interval: 0.76–0.80) [14].

ResR can be defined as the difference between AR and absolute risk reduction (ARR) following intervention. As stated above, the CTT collaboration suggests that a 1 mmol/L decrease in LDL-C is associated with a near 22% relative reduction (rate ratio = 0.78) in CVD risk [14]. Thus, ResR can be estimated from the following equations.

(1) $$RRR=1-0.78^{\alpha }$$

where α is the LDL-C reduction. Figure 1 illustrates the percentage RRR that would be expected using the CTT collaborative rate ratio of 0.78 vs. α (continuous variable) using the above-mentioned equation.

(2) ARR=AR∗RRR

(3) $$ResR=AR-ARR=AR-AR*RRR=AR*\left(1-RRR\right)=AR*0.78^{\alpha }$$

## 4. Association between Age and Absolute Risk

As seen above, ResR post-LDL-C reduction is dependent on AR and LDL-C reduction as seen in Equation (3). It appears clear that any factor will be expected to influence ResR if it is associated with AR. All the risk algorithms calculating AR include age as a predictive variable [3,4,5,6,7,8]. The Whickham Study comprised of 2471 individuals recruited in the Northeast of England between 1972 and 1974 [19]. Age was found to be significantly associated with AR (odds ratio: 1.02, 95% confidence intervals: 1.01–1.04) as was previously demonstrated by analysing the entire Whickham study cohort using a logistic regression model [9]. Age remained significant even when all the other significant risk factors (sex, smoking, systolic blood pressure, diabetes and family history were included in a single logistic regression model). Known measurable CVD risk factors (Table 1) were documented at recruitment and coronary heart disease (CHD) status was recorded as an outcome at the end of the follow-up period of 20 years. Thus, statin therapy was not routinely available (pre 4S [20]) during the follow-up period [19]. In 2000, using the data from the Whickham Study, we confirmed that the AR of CHD in individuals where the Framingham algorithm was applicable (1700 men and women without CVD aged 35–70 years) was only acceptable when the annual AR (observed event rate) for CHD was >1.5% [19]. However, when the AR was <1.5%, the Framingham algorithm underestimated CHD risk [19]. Our results were cited in the 2014 National Institute for Health and Care Excellence (NICE) guidelines; CG 181 (Figure 7 of the document titled Lipid Modification) (https://www.ncbi.nlm.nih.gov/books/NBK248067/pdf/Bookshelf_NBK248067.pdf (accessed on 1 April 2023)). Baseline age was seen to be significantly associated with CHD during the follow-up period of 20 years [9,19].

## 5. Association between Age and Residual Risk Seen in RCTs Using LDL-C Reducing Agents—Trials Included in the CTT Collaboration Stratified by Age

The Whickham Study showed that the probability of CHD over 20 years (AR over 20 years) was positively associated with age [9,19]. Equation (3) states that ResR is positively associated with AR, and thus we can infer that ResR is also positively associated with age. Hence, the older the individual, the greater the ResR. Subgroup analyses of the CTT collaboration by age showed rate ratios (95% confidence intervals) of 0.78 (0.75–0.82), 0.78 (0.74–0.83) and 0.84 (0.73–0.97) for ≤65, >65 to ≤75 and >75 years, respectively [14]. Hence, it appears that in individuals aged > 75 years, in addition to an increasing AR, the RRR would be lower in view of the relative risk of 0.84. This is consistent with the view that the effect of exposure for several decades cannot be simply undone by a few years of cholesterol lowering, and this combination would exaggerate the increase in ResR in older patients.

## 6. Review of Atherogenesis and Cumulative LDL-C Exposure

Ference et al. in 2018 carried out research on the cumulative effect of LDL-C on CVD and the timing of optimizing LDL-C levels [21]. Their paper outlined a cumulative LDL-C exposure threshold that had to be breached for the risk of myocardial infarction to become reality (5000 mg years) and another cumulative LDL-C threshold that was associated with the mean age of developing a myocardial infarction (8000 mg years). They showed that the risk of myocardial infarction increases after the individual breaches the first threshold showing an exponential pattern (linear when the myocardial risk is presented on a log-scale). This scheme suggests that lower LDL-C (either untreated or following LDL-C lowering therapy) would decrease the time related LDL-C exposure, thus reducing the AR of CVD and delay an individual breaching both thresholds. It would be interesting to ascertain whether the thresholds described by Ference et al. would be altered by other risk factors of CVD, e.g., exposure to diabetes, hypertension, smoking, etc. [21]. Nonetheless, it is clear that earlier treatment with LDL-C-reducing therapies (resins, statins, ezetimibe PCSK9 inhibitors, etc.) would reduce ResR by virtue of reducing AR of CVD.

## 7. Possible Reasons That the Rate Ratio of 0.78 Seen in the CTT Collaboration Is Preserved in the Subgroups

It is interesting that LDL-C reduction was significant in all the subgroups in the CTT collaboration with comparable rate ratios and overlapping confidence intervals [14]. This raises the possibility that LDL-C exerts a crucial effect on atherogenesis regardless of the presence or absence of other proven CVD risk factors. For example, current smokers (rate ratio: 0.78, 95% confidence intervals: 0.75–0.82) and non-smokers (rate ratio: 0.78, 95% confidence intervals: 0.73–0.84) had identical rate ratios. This is reassuring as lipid-lowering agents such as statins, ezetimibe, bempedoic acid, PCSK9 inhibitors, inclisiran and resins, either used alone or in combination, offer great efficacy. However, as seen in Figure 1, considerable ResR remains, perhaps owing to other factors, and is also crucial in atherogenesis.

In the above sections, despite demonstrable benefit following LDL-C reduction, there remains significant ResR of CVD. Using published data, we derived mathematical algorithms that would enable an estimation of the RRR, which can be useful to patients considering lipid lowering therapy. Our modelling also shows that ResR is dependent on AR as well as RRR. The rate ratios seen in the CTT collaboration [14] when the cohort was stratified by age hint that ResR may increase in patients > 75 years of age. This review has the potential to help clinicians identify and treat risk factors that may be contributing to the ResR of CVD. In the next section, we will highlight some of these factors.

## 8. Review of Additional Non-Traditional Risk Factors That May Contribute to Residual Risk

Dhindsa et al. in 2020 neatly categorised some of the pathways with trial evidence that could be contributing to ResR [47]. These included inflammatory, thrombotic and metabolic risks; to this aetiology-based stratification we will add endothelial dysfunction.

**a.**  **Potential Inflammatory Risk**

Statins may reduce inflammation (a pleiotropic effect), and a decrease in high sensitivity C-reactive protein was also observed in the JUPITER trial, related to reduced CVD [22]. These findings add some credence to the proposition that inflammatory processes contribute to atherogenesis. However, the association between a reduction in CVD and high sensitivity C-reactive protein was not independent of a decrease in LDL-C [22]. The Pravastatin or Atorvastatin Evaluation and Infection Therapy-Thrombolysis in Myocardial Infarction 22 (PROVE-IT TIMI 22) trial showed that individuals achieving a high sensitivity C-reactive protein < 2 mg/L experienced reduced CVD [23]. The Canakinumab Antiinflammatory Thrombosis Outcome Study (CANTOS) trial studied the impact of 50 mg, 150 mg and 300 mg of canakinumab (which importantly had no effect on LDL-C levels) on CVD risk in patients with a prior myocardial infarction and high sensitivity C-reactive protein values ≥ 2 mg/L [24]. Inexplicably, CVD risk reduction was only significant in patients on 150 mg of canakinumab (as opposed to 50 mg or 300 mg). Further, research is required to move the inflammatory risk pathway from an observed association to causality.

**b.**  **Potential Thrombotic Risk**

There is also some confusion as to the contribution of thrombotic risk to ResR as a benefit following antiplatelet therapy appears restricted to individuals with established CVD as seen in the analyses of 17,999 individuals partaking in 16 RCTs by the Antithrombotic Trialists’ Collaboration; importantly, aspirin was found to reduce serious vascular events [25]. Interestingly, RCTs such as the Prevention of Cardiovascular Events in Patients with Prior Heart Attack Using Ticagrelor Compared to Placebo on a Background of Aspirin–Thrombolysis In Myocardial Infarction 54 (PEGASUS-TIMI 54) and The Effect of Ticagrelor on Health Outcomes in Diabetes Mellitus Patients Intervention (THEMIS-PCI) studies investigating combination treatment of dual antiplatelet treatment of aspirin and ticagrelor (a platelet P2Y12 inhibitor) suggested significant reduction in major adverse cardiac events [26,27]. However, the risk of bleeding with dual antiplatelet therapy was seen to increase.

The combination of the low dose anticoagulant rivaroxaban and antiplatelet therapy was investigated in the Anti-Xa Therapy to Lower Cardiovascular Events in Addition to Standard Therapy in Subjects with Acute Coronary Syndrome–Thrombolysis in Myocardial Infarction Trial 51 (ATLAS ACS 2-TIMI 51) and Cardiovascular Outcomes for People Using Anticoagulation Strategies (COMPASS) trials; the combination treatment was associated with reduced CVD [28,29]. Once again, there was an increased incidence of bleeding.

**c.**  **Potential Metabolic Risk**

There are many factors that could contribute to the metabolic risk that is not addressed by LDL-C reduction. Elevated lipoprotein (a), a particle similar to low density lipoprotein, appears to be a strong predictor of CVD [30]. It is essential to establish whether lipoprotein (a) is just a predictive marker of CVD or actively contributes to atherogenesis. The role of triglycerides and the cholesterol enriched remnant particles of very low-density lipoprotein and chylomicrons are of importance, especially as eicosapentaenoic acid has been recommended in the United Kingdom by NICE (https://www.nice.org.uk/guidance/TA805/chapter/1-Recommendations (accessed on 1 April 2023)). The Reduction of Cardiovascular Events with Icosapent Ethyl–Intervention Trial (REDUCE-IT) showed that eicosapentaenoic acid was associated with lowering triglycerides, CVD and cardiovascular deaths in individuals > 45 years of age with established CVD or individuals > 50 years of age with diabetes and one or more additional CVD risk factor(s) and who had elevated fasting triglyceride levels of 135–499 mg/dL (1.5–5.6 mmol/L), LDL-C at 41–100 mg/dL (1.06–2.6 mmol/L) whilst being on a stable dose of a statin for ≥4 months [31]. It must be stated that the impact of icosapent ethyl on ischemic cardiovascular disease is not predominantly mediated by a reduction of apo B-containing lipoproteins but rather is perhaps multifactorial and not only via a reduction of apo B-containing lipoproteins. 

Although the association between HDL-C levels and atherogenesis has been evident for a long period, there appears to be some confusion, and it will be described briefly. The impact of HDL on ischemic cardiovascular disease is essentially unproven. The matter is complex because low HDL is often a marker of delayed metabolism of triglyceride-rich lipoproteins and of inflammation. The Framingham Heart Study demonstrated an inverse relationship between HDL-C and CVD, but this association was not evident in the Dallas Heart Study where cholesterol efflux as opposed to HDL-C was associated with CVD [4,32]. Further, RCTs with niacin [33] and torcetrapib [34] both elevated HDL-C but failed to decrease CVD. RCTs investigating fibrates, other than gemfibrozil, have presented non-significant CVD outcomes [40,48]. Whilst the Helsinki Heart Study [35] and the Veterans Affairs High-Density Cholesterol Intervention Trial [36], both using gemfibrozil, reduced CVD, this was not observed in the three subsequent studies not using gemfibrozil (Bezafibrate Infarction Prevention study [37], Fenofibrate Intervention and Event Lowering in Diabetes [38] and Action to Control Cardiovascular Risk in Diabetes–LIPID [39]). However, in a subgroup of individuals with dyslipidaemia characteristic of the metabolic syndrome, lower CVD appeared associated with fibrate therapy. Bruckert et al. showed a statistically significant reduction of 28% in CVD in individuals with HDL-C and triglyceride values closest to those of the metabolic syndrome (HDL-C < 0.91 mmol/L and triglycerides > 2.2 mmol/L in the above trials [40]. In contrast, the complementary group showed only a non-significant 6% risk reduction. Further, a meta-analysis of 18 RCTs with fibrate therapy by Jun et al. demonstrated significant reduction (rate ratio: 0.87, 95% confidence intervals: 0.81–0.93) of CVD [49].

Two of the newer classes of drugs, glucagon like peptide 1 receptor agonists (GLP-1RA) and sodium-glucose cotransporter 2 (SGLT2) inhibitors, used in type 2 diabetes treatment, have been seen to reduce ResR [41,50,51,52,53,54]. Interestingly in the Empagliflozin, Cardiovascular Outcomes, and Mortality in Type 2 Diabetes (EMPAREG-OUTCOME) trial a high proportion of patients were on antihypertensives, statins and aspirin, despite that ResR was high in the placebo group [41]. Further study of potential anti-atherogenic mechanisms of these 2 drug classes must be conducted to develop further strategies that lower ResR.

**d.**  **Potential Risk Associated with Endothelial Dysfunction**

Another interesting factor that could potentially contribute to atherogenesis is endothelial dysfunction [55]. Atherogenesis is associated with vessel wall injury and other local and systemic risk factors and appears to be related to reduced nitric oxide synthase synthesis, which leads to altered arterial wall shear stress, vasodilation and cell repair [56,57]. Traditional CVD risk predictors such as diabetes, dyslipidaemia, smoking and hypertension lead to endothelial cell dysfunction [58]. The Heart Outcomes Prevention Evaluation (HOPE) [42,43], Captopril Prevention Project (CAPP) [44] and Appropriate Blood Pressure Control in Diabetes (ABCD) [45] RCTs suggested that antihypertensives reduced CVD, with this decrease often exceeding the risk reduction that could be attributed to the lowering of blood pressure. Further, longitudinal cohort studies have demonstrated that Phosphodiesterase type 5-inhibitors reduce risk of both myocardial infarction [59] and all-cause mortality [60,61].

In view of this, our group compared differences in blood flow data and computational flow dynamics in 27 subjects with established CHD and 30 individuals without any symptoms of CHD [46]. Our analyses hinted that peak systolic velocity may be a predictive factor of CHD; despite the modest cohort numbers, a significant difference was observed (patients without CHD, mean (SD): 62.8 (16.1) cm/s, patients with CHD, mean (SD): 53.6 (17.3) cm/s, *p* = 0.042). Further, factors such as wall shear stress were associated with peak systolic velocity [61]. We speculated that peak systolic velocity could be a composite surrogate factor as it may be associated with many of the risk factors altering atherogenicity of the vessel wall.

The impact of all these potential risk factors may be additive and cumulative and thus age related. The combination of these and other undetermined risk mechanisms associated with age may lead to increased AR in older individuals and, as shown above, higher ResR as well.

## 9. Discussion

**Figure 2:**
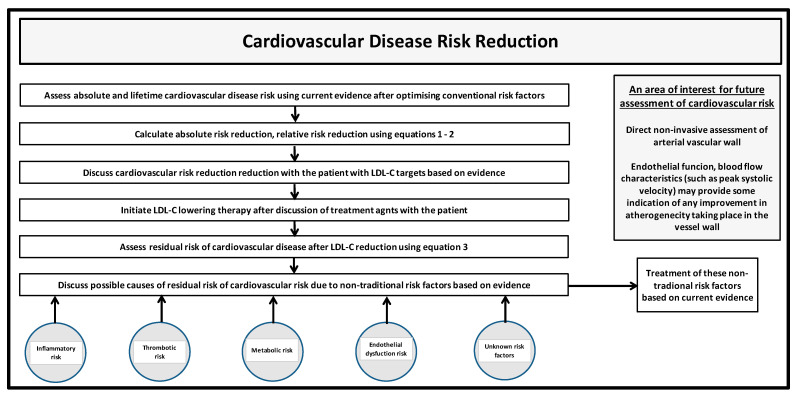
A proposal for the discussion of CVD risk, risk reduction, ResR and possible causes between the health care professional and the patient.

All CVD risk algorithms have age as a significant risk factor. The various RCTs have shown ResR to be significant despite significantly lowering LDL-C. We have explored the relationship between RRR, ARR, AR and ResR using mathematical equations and have demonstrated that ResR was a function of AR and LDL-C reduction. We also recognise that the performance of risk prediction models entails discrimination and calibration; hence, all models are limited. Further, we are aware that whilst interventions to some extent, delay events and deaths, they can never prevent death itself. We have also identified several possible non-traditional risk factors that may contribute to ResR and discussed RCTs providing some evidence for future treatments. However, no clear consensus exists at this moment for these interventions to be included in guidelines. Data from further research will determine whether therapies addressing non-traditional risk factors will be used in addition to LDL-C lowering agents. Figure 2 outlines a practical proposal for the discussion of CVD risk, risk reduction, ResR and possible causes between the health care professional and the patient.

There is some evidence that the lowering of apo B (found in LDL, intermediate density lipoprotein (IDL) and very low-density lipoprotein (VLDL) particles) may be better than LDL-C in risk assessment models. In 2019 the European Society of Cardiology and the European Atherosclerosis Society, using data from prospective observational studies, Mendelian randomisation studies and statin trials meta-analyses, suggested that apo B is a more predictive marker than LDL-C of CVD risk and treatment efficacy to reduce cardiovascular risk [62]. It is perhaps even better in individuals with hypertriglyceridaemia, obesity, diabetes and the metabolic syndrome where high levels of VLDL and IDL particles may also contribute to CVD risk in addition to LDL particles [63]. Prior to routine clinical use and inclusion in guidelines, standardisation of apo B assays is essential. Currently, apo B assays are both accurate and precise over wide concentration levels and take into account LDL and the other atherogenic lipoprotein particles [63]. There will be cost implications for healthcare systems to switch over from LDL-C to apo B as a risk predictor. There would have to be more research to establish rate ratios per unit reduction in apo B such as was evident in the CTT collaboration [14]. Thus, it is our clinical practice to measure apo B in patients with high triglyceride levels to provide additional information to that offered by LDL-C. However, we are aware that Equations (1)–(3) can be adapted easily from LDL-C to apo B reduction with appropriate rate ratios.

Heterogeneity in presentation and outcomes following treatment is common in patients with chronic diseases [64]. Regression of atheroma may be at odds with ResR calculation, which can never reach 0% as evident from Equation (3). The ASTEROID study using rosuvastatin 40 mg/d for 24 months achieved an average LDL-C of 1.57 mmol/L (60.8 mg/dL) and resulted in significant regression of atherosclerosis in many individuals for prespecified intravascular ultrasound (IVUS) measures of atheroma [65]. Similar results were observed in the SATURN study in individuals on atorvastatin 80 mg and rosuvastatin 40 mg after 104-week s of treatment [66]. Mean LDL-C values at study end were 1.82 mmol/L (70.2 mg/dL) and 1.62 mmol/L (62.6 mg/dL) in the atorvastatin and rosuvastatin study arms respectively. Regression was observed in percent atheroma volume (63.2% and 68.5% of patients on atorvastatin and rosuvastatin respectively) and total atheroma volume (64.7% and 71.3% of patients on atorvastatin and rosuvastatin respectively) on IVUS [66]. It must be emphasized that the ASTEROID and SATURN studies used IVUS measurements as outcomes and not hard endpoints as in the CTT Collaboration. Thus, it is essential that association between atheroma plaque progression/regression is studied in detail as suggested by Dawson et al. in 2022 [67]. They suggested that in the event of outcome data being associated with plaque regression, monitoring coronary plaque may replace surrogate markers like CVD risk algorithms and lipid/lipoprotein levels.

As mentioned previously, we have shown lower peak systolic velocity via non-invasive portable ultrasound equipment was associated with coronary artery disease [46]. It is also important that associations between markers such as peak systolic velocity, IVUS parameters and major adverse cardiovascular events are established before they can be used to address outcome heterogeneity following lipid lowering and other CVD risk reduction therapies. It is only at that point that markers such as peak systolic velocity can be offered routinely. All the main studies covered in this review are stratified by topic and summarised in Table 1.

## 10. Conclusions

As ResR is a function of AR and LDL-C reduction, we can conclude that in addition to an increasing AR, the RRR would be lower in view of a higher relative risk in individuals aged > 75 years. This supports the adopted understanding that the effect of exposure for several decades cannot be simply undone by a few years of cholesterol lowering treatment. This, together with an exaggerated increase in ResR in older patients, underlines the need for early intervention, be that lifestyle or therapy. The included Figure 1 demonstrating ResR could be used by healthcare professionals to discuss CHD risk and the benefits of lifetime risk reduction as opposed to ARR. Whilst we discussed the non-LDL-C risk factors such as inflammation, thrombosis, metabolic and endothelial status, and the potential cardiovascular benefits of improving these with therapeutic agents, future efforts should be directed towards aiding clinicians to identify and treat risk factors that may be contributing to the ResR of CVD. The novel elements of our work used mathematical modelling to demonstrate to clinicians and patients the ResR post LDL-C reduction to facilitate recognition of the potential factors associated with ResR to be addressed. Our system can be extended with the addition of future models estimating non-LDL-C based relative risk reduction. We hope that this paper may help professionals to further understand ResR and also emphasise the importance of non-lipid lowering agents such as eicosapentaenoic acid, SGLT2 inhibitors and GLP1RA that are currently available in the United Kingdom.

## Figures and Tables

**Table 1:** A brief description of the principal studies described in this review.

	Evidence	Main Finding	Reference
**LDL-C reduction**	Review of RCTs by Ramachandran et al.	Validity of the LDL-C (lipid) hypothesis	[9]
	CTT Collaboration (review of 26 RCTs using statins)	Establishing a relative CVD risk reduction of 22% per mmol/L decrease in LDL-C	[14]
	RCTs using Ezetimibe	CVD decrease in line with the CTT Collaboration	[15,16]
	RCTs using Proprotein Convertase Subtilisin/Kexin Type-9 inhibitors		[17,18]
**Residual Risk**	Rate ratio of 0.78 from the CTT Collaboration	Calculation of RRR, ARR and ResR	[14]
**Age**	Framingham Heart Study	Age is a significant risk factor in CVD predictive algorithms	[3,4]
	PROCAM Study		[5]
	SCORE Project		[6,7]
	QRISK		[8]
	Whickham Study		[19]
**LDL-C exposure**	Analysis of trials by Ference et al.	Cumulative LDL-C exposure is related to CVD	[21]
**Inflammatory Risk**	JUPITER RCT	Decrease in high sensitivity C-reactive protein was associated with lower CVD	[22]
	PROVE-IT TIMI 22 RCT		[23]
	CANTOS RCT	Moderate dose (not lower or higher) of canakinumab was associated with lower CVD	[24]
**Thrombotic Risk**	Antithrombotic Trialists’ Collaboration RCT	Aspirin reduced CVD	[25]
	PEGASUS-TIMI 54 RCT	Combination of spirin and ticagrelor therapy reduced CVD	[26]
	THEMIS-PCT RCT		[27]
	ATLAS ACS 2-TIMI 51 RCT	Combination of rivaroxaban and antiplatelet therapy reduced CVD	[28]
	COMPASS RCT		[29]
**Metabolic Risk**	INTERHEART Study	High lipoprotein (a) levels associated with increased CVD	[30]
	REDUCE-IT RCT	Eicosapentanoic acid associated with lowering of CVD	[31]
	Dallas Heart Study	HDL-C levels were not associated with CVD	[32]
	AIM-HIGH RCT	HDL-C increase following niacin treatment was not associated with CVD decrease	[33]
	ILLUMINATE RCT	HDL-C increase following torcetrapib treatment was not associated with CVD decrease	[34]
	Helsinki Heart Study	Gemfibrozil treatment was associated with lower CVD	[35]
	Veterans Affairs High-Density Cholesterol Intervention Trial		[36]
	Bezafibrate Infarction Prevention study	Bezafibrate treatment was not associated with lower CVD	[37]
	Fenofibrate Intervention and Event Lowering in Diabetes	Fenofibrate treatment was not associated with lower CVD	[38]
	Action to Control Cardiovascular Risk in Diabetes—LIPID		[39]
	Analysis by Bruckert et al. of fibrate RCTs	CVD was significantly lower in patients with high triglycerides and low HDL-C	[40]
	EMPA-REG OUTCOME RCT	Empagliflozin treatment reduced CVD	[41]
**Endothelial Dysfunction**	HOPE RCT	Antihypertensives reduced CVD	[42,43]
	CAPP RCT		[44]
	ABCD RCT		[45]
	König et al. case-control study	Lower peak systolic velocity was associated with CHD	[46]
